# Comment on ‘Factors associated with depressive symptoms among adolescents in Indonesia: A cross-sectional study of results from the Indonesia Family Life Survey’

**DOI:** 10.51866/lte.26-00019

**Published:** 2026-08-29

**Authors:** Esra Rabia Taşpolat

**Affiliations:** 1 Department of Child and Adolescent Psychiatry, Iğdır Dr. Nevruz Erez State Hospital, Iğdır, Türkiye.

**Keywords:** Adolescent, Depressive symptoms, Chronic disease, Mental health, Primary health care

**Dear Editor,**

I read with great interest the article by Idris and Tuzzahra examining the factors associated with depressive symptoms among Indonesian adolescents using data from the Indonesia Family Life Survey.^1^ The authors reported that chronic illness history was the strongest factor associated with depressive symptoms, followed by female sex, poor sleep quality, smoking, low socioeconomic status and introverted personality. Their findings provide valuable insights into the epidemiology of adolescent depression in a large nationally representative sample. I would like to offer several comments that may further strengthen the interpretation of these findings.

First, the conclusion that chronic illness is the most influential factor associated with depressive symptoms warrants careful consideration. The chronic illness variable encompassed a broad range of medical conditions, including asthma, tuberculosis, cardiovascular diseases, kidney diseases, gastrointestinal disorders and cancer. These conditions vary considerably in severity, functional impact, treatment burden and psychosocial consequences.^2,3^ Consequently, grouping them into a single category may obscure important differences in their relationships with depressive symptoms. Future studies examining illness-specific associations may provide a more clinically meaningful understanding of risks.

Second, several well-established determinants of adolescent depression were not included in the analyses. Family psychiatric history, parental depression, adverse childhood experiences, family functioning, peer relationships and social support have consistently been identified as important contributors to adolescent mental health outcomes.^4^ The absence of these variables raises the possibility of residual confounding and may limit the interpretation of the independent contribution of chronic illness to depressive symptoms.

Finally, the identification of chronic illness history as the strongest correlate of depressive symptoms is clinically compelling and consistent with international evidence. However, the cross-sectional design of this study precludes any causal inference. Adolescent depression itself may contribute to health-risk behaviours – such as smoking, poor sleep hygiene and reduced physical activity – which consequently increase susceptibility to chronic conditions. The bidirectional nature of this association is well documented in the literature.^4,5^ The authors appropriately note this as a limitation, but future research employing longitudinal or prospective cohort designs is urgently needed to disentangle these temporal relationships, particularly in the Indonesian context.

Despite these limitations, the study carries important implications for clinical practice and public health. Adolescents living with chronic medical conditions constitute a particularly vulnerable group for emotional and behavioural difficulties. The relationship between chronic illness and depression appears to be bidirectional. Longitudinal evidence suggests that depressive symptoms in adolescents with chronic illnesses predict poorer disease control, reduced treatment adherence, increased hospitalisation rates, greater disability and lower quality of life over time.^3^ Therefore, depression should be viewed not only as a psychological consequence of chronic disease but also as a factor that may adversely influence the course and management of chronic medical conditions.

These findings are particularly relevant to primary care physicians, who are often the first health care professionals to identify both chronic medical conditions and emerging mental health difficulties in adolescents. Therefore, primary care settings may provide a valuable opportunity for the early detection of depressive symptoms and timely intervention. The findings support the development of proactive mental health screening strategies for high-risk youth. School-based mental health screening programmes may facilitate the early recognition of depressive symptoms before they progress to more severe disorders. In addition, routine depression screening within chronic disease clinics – including paediatric, endocrinology, pulmonology, neurology and other specialty services – could improve the identification of adolescents who might otherwise remain undiagnosed. Integrating mental health assessment into routine medical care may enable earlier referral, timely intervention and improved long-term outcomes.

In summary, I believe that the findings of Idris and Tuzzahra further support the strengthening of preventive mental health services through early detection initiatives, school-based screening programmes and integrated care models for adolescents with chronic illnesses. Future longitudinal studies incorporating family, psychosocial and illness-specific factors may help clarify the mechanisms underlying the relationship between chronic disease and depressive symptoms during adolescence.
